# Adenoviruses-mediated transduction of human oesophageal carcinoma cells with the interferon-*λ* genes produced anti-tumour effects

**DOI:** 10.1038/bjc.2011.379

**Published:** 2011-09-27

**Authors:** Q Li, K Kawamura, S Okamoto, H Fujie, M Numasaki, M Namba, M Nagata, H Shimada, H Kobayashi, M Tagawa

**Affiliations:** 1 Division of Pathology and Cell Therapy, Chiba Cancer Center, Chiba 260-8717, Japan; 2 Department of Molecular Biology and Oncology, Graduate School of Medicine, Chiba University, Chiba 260-8670, Japan; 3 Department of Biochemistry, Graduate School of Pharmaceutical Science, Chiba University, Chiba 260-8675, Japan; 4 Department of Nutritional Physiology, Faculty of Pharmaceutical Science, Josai University, Sakado 350-0295, Japan; 5 Niimi College, Okayama 718-8585, Japan; 6 Division of Gastroenterological Surgery, Chiba Cancer Center, Chiba 260-8717, Japan; 7 Department of Surgery, School of Medicine, Toho University, Tokyo 143-8540, Japan

**Keywords:** adenovirus, IFN-*λ*, oesophageal carcinoma, apoptosis, gene therapy

## Abstract

**Background:**

Interferon-*λ*s (IFN-*λ*s) are novel cytokines with multiple functions, like IFN-*α* and -*β*. We examined possible anti-tumour effects produced by adenoviruses bearing the *IFN-λ1* or *-λ2* gene (Ad/IFN-*λ*) with the type-35 fibre-knob structure.

**Methods:**

Proliferation of oesophageal carcinoma cells transduced with Ad/IFN-*λ* and mechanisms of the inhibited growth were investigated.

**Results:**

Transduction with Ad/IFN-*λ* upregulated the expression of the class I antigens of the major histocompatibility complexes and induced the growth suppression. Increased sub-G1 populations and the cleavage of caspase-3 and poly (ADP-ribose) polymerase were detected in IFN-*λ*-sensitive YES-2 and T.Tn cells. The cell death was accompanied by cytoplasmic cytochrome *C* and increased cleaved caspase-9 and Bax expression, suggesting mitochondria-mediated apoptosis. Adenovirus/IFN-*λ*-infected YES-2 cells subsequently reduced the tumourigenicity. Adenovirus/IFN-*λ*-infected fibroblasts, negative for the IFN-*λ* receptors, induced death of YES-2 or T.Tn cells that were co-cultured. Inoculation of YES-2 cells in nude mice, when mixed with the Ad/IFN-*λ*-infected fibroblasts, resulted in retardation of the tumour growth. The growth suppression was not linked with upregulated CD69 expression on natural killer cells or increased numbers of CD31-positive cells.

**Conclusion:**

Adenovirus/IFN-*λ* induced apoptosis, and fibroblast-mediated delivery of IFN-*λ*s is a potential cancer treatment by inducing direct cell death of the target carcinoma.

Interferon-*λ*s (IFN-*λ*s) belong to type III IFN that consists of IFN-*λ*1, -*λ*2 and -*λ*3, which are also known as interleukin (IL)-29, -28A and -28B, respectively (Kotenko *et al*, 2003; Sheppard *et al*, 2003). A novel IL-28 receptor-*α* (IL-28R*α*) constitutes the heterodimeric IFN-*λ* receptor complexes with the IL-10 receptor-*β* (IL-10R*β*), and the receptor complexes bind all the IFN-*λ* subtypes (Sheppard *et al*, 2003). Binding of respective ligands to the receptors activates the Janus kinases and induces the STAT1 and the STAT2 phosphorylation, like type I IFNs, IFN-*α* and IFN-*β*, although the structures of the type I and III receptor complexes are different (Pestka *et al*, 2004). Activation of the signal transduction systems results in formation of the IFN-stimulated regulatory factor 3 complexes and subsequently initiates transcription of IFN-stimulate genes (Kotenko *et al*, 2003; Li *et al*, 2009). Type I and III IFNs share some of the signal transduction pathways but further analyses showed their differential responsiveness to virus infections (Marcello *et al*, 2006), suggesting discrete biological significances between the two IFN families.

Interferons have multiple biological activities, including anti-viral, immunomodulatory and anti-proliferative actions (Pestka *et al*, 2004). The IFN-*λ*s as well as the type I IFNs produce anti-viral molecules, such as myxovirus resistance A and 2′,5′-oligoadenylate synthetase, and inhibit viral replications, but the repertoires of target viruses could be different from those of type I IFNs (Li *et al*, 2009). The immunological activities of IFN-*λ*s have been investigated to show that IFN-*λ*s upregulated expression levels of the class I molecules of the major histocompatibility complexes (MHC; Kotenko *et al*, 2003; Li *et al*, 2009), and that local secretion of IFN-*λ*2 from tumours enhanced cell-mediated immunity against the tumours by augmenting activities of natural killer (NK) cells and cytotoxic T cells (Sato *et al*, 2006; Numasaki *et al*, 2007). These immunological actions of IFN-*λ*s are thus similar to those of type I IFNs. In contrast, IFN-*λ*s-mediated growth suppression has not yet well explored. Previous reports merely showed the growth inhibitory action in several tumour cells, including a neuroendocrine tumour, a glioblastoma and colon cancer cell lines (Brand *et al*, 2005; Meager *et al*, 2005; Zitzmann *et al*, 2006).

Oesophageal carcinoma is one of intractable diseases, as it frequently develops in aged persons. Extensive surgery decreases the patients’ quality of life, and radical chemotherapy and radiotherapy are not often appropriate for the aged patients. A novel therapeutic strategy is thereby required to improve the prognosis. Gene therapy with adenoviruses (Ad) expressing a therapeutic gene is a possible approach, and IFN can be one of the molecules to be tested as recombinant IFN-*α* (rIFN-*α*) has been clinically investigated for the efficacy in various types of malignancy including oesophageal carcinoma (Parmar and Platanias, 2003). Adenovirus-mediated gene transfer of IFN-*β* was also examined for the clinical feasibility (Yoshida *et al*, 2004), but type I IFNs produced a number of adverse effects (Dusheiko, 1997), which may limit the extensive clinical applications. In contrast, a few animal studies have implied IFN-*λ*s as a possible anti-cancer agent (Lasfar *et al*, 2006; Sato *et al*, 2006; Numasaki *et al*, 2007), and none of clinical studies has been performed with IFN-*λ*s.

We examined the transduction efficacy of Ad with a panel of human oesophageal carcinoma cells and found that type 5 Ad bearing the type-35-derived fibre-knob region infected them better than type 5 Ad (Yu *et al*, 2005), as the expression levels of type 5 receptors, coxsackie Ad receptors, are often downregulated but those of type 35 receptors, CD46, are well maintained in human tumours. Moreover, we also recently showed that rIFN-*λ*1 inhibited the growth of human oesophageal carcinoma by inducing G1 arrest or apoptosis, depending on the cells tested (Li *et al*, 2010). In this study, we produced fibre-knob modified type 5 Ad expressing *IFN-λ1* or *IFN-λ2* gene, investigated whether Ad-mediated expression of IFN-*λ*s could suppress the growth of oesophageal carcinoma cells and tested a possible gene therapy with the Ad and the Ad-infected fibroblasts.

## Materials and methods

### Cells

Human oesophageal carcinomas TE-1, TE-2, TE-10, TE-11, YES-2, YES-4, YES-5, YES-6 and T.Tn cells, human fibroblasts OUMS-24, OUMS-24/P6X (P6X; Fushimi *et al*, 1997) and HFF (Compton, 1993), and human embryonic kidney 293 cells were cultured in RPMI-1640 medium supplemented with 10% fetal calf serum. Human immortalised, but not transformed, Het-1A cells of oesophageal epithelial origin were purchased from American Type Culture Collection (Manassas, VA, USA) and were maintained with a specified medium as recommended by the suppliers.

### Construction of Ad vector

The *green fluorescent protein* (GFP), *β-galactosidase* (LacZ), human *IFN-λ1* and *IFN-λ2* genes were cloned into pShuttle 2 (Takara Bio, Tokyo, Japan) and then ligated with Adeno-X vector (Takara Bio), of which the fibre-knob region was replaced with that of type 35 Ad. The fibre-knob modified Ad DNA was produced by inserting the *EcoR*I fragment containing the type 35 Ad fibre-knob region (Avior Therapeutics, Seattle, WA, USA; AY271307 at 30827-33609) into the corresponding site of Adeno-X vector DNA. The modified Ad expressing the above genes, Ad/GFP, Ad/LacZ, Ad/IFN-*λ*1 and Ad/IFN-*λ*2, were produced by transfecting the respective DNA into 293 cells and purified with an Adeno-X virus purification kit (BD Biosciences, San Jose, CA, USA).

### Infectivity of Ad

Cells were infected with Ad/GFP at multiplicity of infection (MOI) of 3 or 30 for 30 min and were washed to remove Ad. Infected cells were cultured for 2 days and then analysed for percentages of GFP-positive cells with FACSCalibur (BD Biosciences) and CellQuest software (BD Biosciences). Cells whose fluorescence was greater than the brightest 5% of uninfected cells were judged as positively stained.

### Flow cytometry and cell cycle analysis

Cells were stained with fluorescein isothiocyanate (FITC)-conjugated anti-HLA-A/B/C antibody (Ab) or FITC-conjugated isotype-matched control Ab (BD Biosciences) or anti-CD90 Ab (Calbiochem, Darmstadt, Germany), followed by FITC-conjugated goat anti-mouse Ab (SouthernBiothech, Birmingham, AL, USA) and propidium iodide (PI, 5 *μ*g ml^−1^). They were then analysed for their fluorescence intensity with FACSCalibur and CellQuest software. For cell cycle analysis, cells were treated with ice-cold 70% ethanol and then with RNase (50 *μ*g ml^−1^), and were stained with PI (50 *μ*g ml^−1^). Cells were also reacted with anti-proliferation cell nuclear antigen (PCNA) Ab (Santa Cruz Biotechnology, Santa Cruz, CA, USA), and the immunohistochemical stainings were visualised with a staining kit (Nichirei Biosciences, Tokyo, Japan).

### Cell proliferation and viability

Cells were treated with Ad, human rIFN-*λ*1 or rIFN-*λ*2 (R&D Systems, Minneapolis, MN, USA), and viable cell numbers were counted on the basis of the trypan blue dye exclusion test. The viabilities were assessed with a WST-8 reagent (Dojindo, Kumamoto, Japan), and the amounts of formazan produced were determined with the absorbance at 450 nm (WST assay). The relative viability was calculated on the basis of the absorbance without any treatments.

### Western blot analysis and enzyme-linked immunosorbent assay (ELISA)

Cells lysates prepared with a protease inhibitor cocktail (Sigma-Aldrich, St Louis, MO, USA) were subjected to SDS–polyacrylamide gel electrophoresis and probed with anti-caspase-3, anti-cleaved caspase-3, anti-poly (ADP-ribose) polymerase (PARP), anti-cleaved PARP, anti-caspase-8, anti-cleaved caspase-8, anti-caspase-9, anti-cleaved caspase-9, anti-cytochrome *C*, anti-Bax and anti-Bcl-2 Ab (Cell Signaling, Beverly, MA, USA). The membranes were developed with the ECL system (GE Healthcare, Buckinghamshire, UK), and the same filter was re-probed with anti-actin Ab (Sigma-Aldrich) or anti-glyceraldehyde-3-phosphate dehydrogenase (GAPDH) Ab for a loading control. For cytochrome *C* expression, a cytoplasmic fraction was purified (Clohessy *et al*, 2006) and the GAPDH expression was used as a control. Secreted IFN-*λ*s in culture supernatants were subjected to immunoblotting with anti-IFN-*λ*1 or anti-IFN-*λ*2 Ab (R&D Systems). The amounts of secreted IFN-*λ*s and the concentrations *in vivo* were determined by an ELISA kit (for IFN-*λ*1, eBioscience, San Diego, CA, USA; for IFN-*λ*2, R&D Systems).

### Animal experiments, immunohistochemical staining and NK cell markers

YES-2 cells (1 × 10^6^), uninfected or infected with Ad, or mixed populations of YES-2 cells (1 × 10^6^) and P6X cells (1 × 10^6^), uninfected or infected with Ad, were injected subcutaneously into BALB/c *nu*/*nu* mice (6-week-old females, Japan SLC, Hamamatsu, Japan). Tumour volume was calculated according to the formula (1/2 × length × width^2^). Tumour tissues were immediately frozen on day 21 after the inoculation and the 4-*μ*m sections were incubated with anti-CD31 Ab (BD Biosciences), followed by peroxidase-conjugated anti-rat Ab (Nichirei Biosciences) and 3,3′-diaminobenzidine (Nichirei Biosciences). Spleen cells of naïve or tumour-bearing mice on day 10 were stained with FITC-conjugated anti-NK cell Ab (DX5, BD Biosciences) and phycoerythrin (PE)-conjugated anti-CD69 Ab (eBioscience). Staining profiles were analysed with FACSCalibur and CellQuest software. All the animal experiments were approved by the animal experiment and welfare committee at Chiba Cancer Center Research Institute.

### Reverse transcription PCR (RT-PCR) and PCR analysis

Human specimens were obtained from the Chiba Cancer Center Biobank, and the use for this research had been approved by the Chiba Cancer Center Ethical Committee. First-strand cDNA was synthesised with Superscript III reverse transcriptase (Invitrogen, Carlsbad, CA, USA), and amplification of equal amounts of the cDNA was performed with the following primers and conditions: for the *IL-28Rα* gene, 5′-GGGAACCAAGGAGCTGCTATG-3′ (sense), 5′-TGGCACTGAGGCAGTGGTGTT-3′ (anti-sense) and 10 s at 94 °C for denature/20 s at 58 °C for annealing/28 cycles; for the *IL-10Rβ* gene, 5′-TATTGGACCCCCTGGAAT-3′ (sense), 5′-GTAAACGCACCACAGCAA-3′ (anti-sense) and 10 s at 94 °C/20 s at 50 °C/28 cycles; for the *CD90* gene, 5′-AACGGCCTGCCTAGTGGAC-3′ (sense), 5′-CCAGAGGTGTGCGGAGAG-3′ (anti-sense) and 30 s at 94 °C/30 s at 59 °C/35 cycles; for the *monokine induced by IFN-γ* (*Mig*) gene, 5′-GACATTCTCGGACTTCACTC-3′ (sense), 5′-GATTCAGGGTGCTTGTTGGT-3′ (anti-sense) and 15 s at 95 °C/15 s at 55 °C/25 cycles; and for the *GAPDH* gene, 5′-ACCACAGTCCATGCCATCAC-3′ (sense) 5′-TCCACCACCCTGTTGCTGTA-3′ (anti-sense) and 15 s at 94 °C/15 s at 60 °C/25 cycles. Amplification of genomic DNA of tumours was performed as follows: for the type 35 Ad-derived fibre-knob region, 5′-TTTCTTACTCCTCCCTTTGTATCCC-3′ (sense), 5′-AATTGAAAAATAAACACGTTGAAAC-3′ (anti-sense) and 10 s at 95 °C/30 s at 55 °C/35 cycles; for the *IFN-λ2* gene, 5′-CCTCAGAGTGTTTCTTCTGC-3′ (sense), 5′-CACAATGCTTCCATCAAACG-3′ (anti-sense) and 10 s at 94 °C/20 s at 55 °C/30 cycles; and for the *GAPDH* gene, 5′-ACCACAGTCCATGCCATCAC-3′ (sense), 5′-TCCACCACCCTGTTGCTGTA-3′ (anti-sense) and 15 s at 94 °C/15 s at 60 °C/23 cycles.

## Results

### IFN-*λ* secretion from Ad/IFN-*λ*-infected oesophageal carcinoma cells

**Figure 1:**
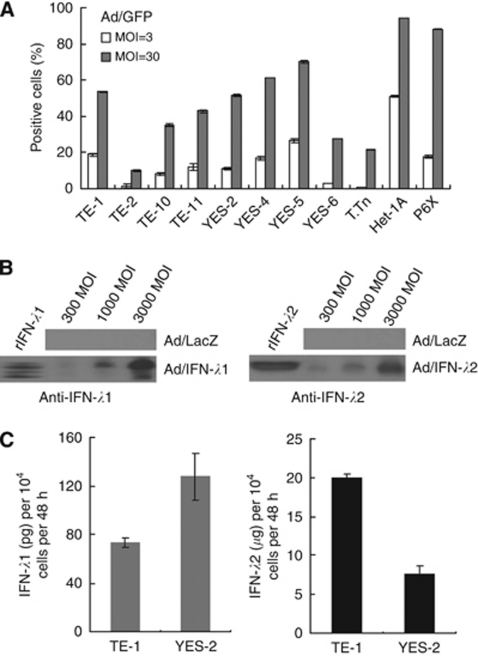
Infectivity of fibre-knob-modified Ad and IFN-*λ* secretion. (**A**) Flow cytometrical analyses of GFP^+^ oesophageal carcinoma, Het-1A and P6X cells that were infected with Ad/GFP. Mean percentages and s.e. bars are shown (*n*=3). (**B**) Western blot analyses of IFN-*λ*1 and IFN-*λ*2 in cell-free culture supernatants. YES-2 cells were cultured for 48 h following Ad infection with different MOIs, and an aliquot was subjected to electrophoresis with rIFN-*λ*1 or rIFN-*λ*2 as a control. (**C**) Secreted amounts of IFN-*λ*1 or IFN-*λ*2 from cells infected with Ad/IFN-*λ*1 or Ad/IFN-*λ*2 at 1000 MOI were assayed with an ELISA. The s.e. bars are also shown (*n*=3).

We used Ad/GFP with the type-35-derived fibre-knob region, which showed better transduction efficacy than type 5 Ad, and examined the transduction levels of nine kinds of oesophageal carcinoma cells, oesophageal epithelial Het-1A cells and P6X fibroblasts (Figure 1A). All of them were infected, and the percentages of GFP^+^ cells varied among the cells, which could reflect Ad receptor expression levels and transcriptional activities of the cytomegalovirus promoter that activated the *GFP* gene. We next examined secretion of IFN-*λ*1 and IFN-*λ*2 from Ad/IFN-*λ*1- and Ad/IFN-*λ*2-infected cells, respectively, with western blot analyses and an ELISA. We detected both IFN-*λ*s in the supernatants of Ad-infected YES-2 cells in a MOI-dependent manner (Figure 1B) and found that the secreted amounts of IFN-*λ*2 were greater than those of IFN-*λ*1 in both TE-1 and YES-2 cells, which were infected at the same MOI (Figure 1C). The reasons for the differential IFN-*λ* production are currently unknown. Adenovirus/LacZ-infected cells did not secrete any detectable amounts of IFN-*λ*s, suggesting Ad infection itself did not produce IFN-*λ*s.

### Growth suppressive activity of Ad/IFN-*λ*

**Figure 2:**
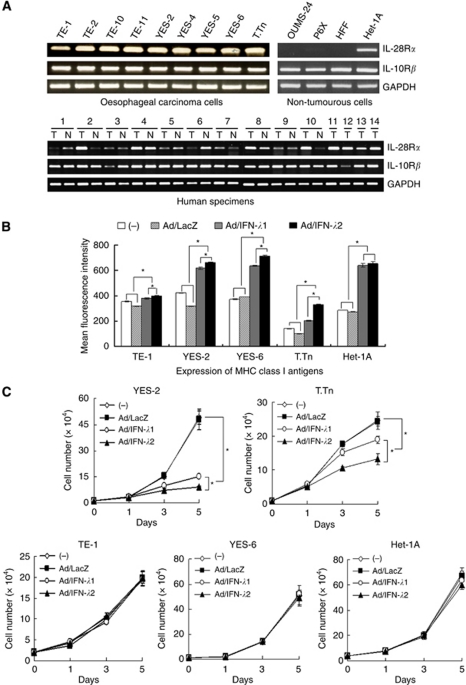

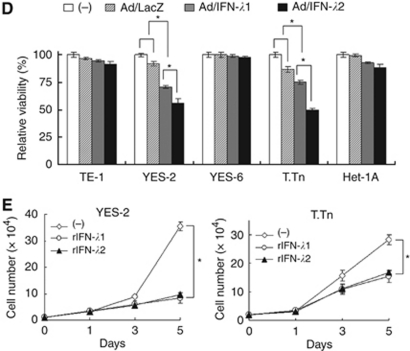
Adenovirus/IFN-*λ*-mediated growth suppression. (**A**) Expressions of the *IL-28Rα* and the *IL-10Rβ* genes were analysed with RT–PCR. Paired 10 specimens from oesophageal tumours (T) and non-tumourous regions of the same patients (*N*), and 4 additional tumours samples were also tested. The *GAPDH* expression was shown as a control. (**B**) Cells were infected with Ad (1000 MOI) and cultured for 3 days. Mean fluorescence intensity of MHC class I antigens was analysed with flow cytometry. The s.e. bars are also shown (*n*=3). (**C**) Cells were treated with or without Ad (1000 MOI) and then live cell numbers were counted. Means and s.e. bars are shown (*n*=3). (**D**) Cells were treated with Ad (1000 MOI), and the percent relative viabilities were measured with the WST assay on day 5. Means and s.e. bars are shown (*n*=3). (**E**) Proliferation of cells treated with rIFN-*λ*1 or rIFN-*λ*2 (100 ng ml^−1^). Means and s.e. bars are shown (*n*=3). ^*^P<0.01.

We examined the expression of IFN-*λ* receptor complexes in oesophageal carcinoma cells, Het-1A and fibroblasts OUMS-24, P6X and HFF cells (Figure 2A). Both the *IL-28Rα* and the *IL-10Rβ* genes were expressed in all the oesophageal carcinoma and Het-1A cells, but the *IL-28Rα* expression was undetectable in the fibroblasts, suggesting that the receptor expression is restricted in a tissue-specific manner. We also examined the expression in clinical specimens of oesophageal carcinoma and a non-tumourous oesophageal region of the same patients (Figure 2A). Both the *IL-28Rα* and the *IL-10Rβ* genes were expressed in all the tumour and non-tumourous specimens, but expression levels of the *IL-28Rα* gene were variable among the specimens. The paired 10 samples did not show any preferential elevation of the *IL-28Rα* gene in carcinoma samples. We examined whether infection with Ad/IFN-*λ*1 or Ad/IFN-*λ*2 upregulated expression levels of the MHC class I molecules, and flow cytometrical analyses showed that the transduction increased the class I expression in oesophageal carcinoma and Het-1A cells, although the enhanced levels were variable among the cells (Figure 2B). We tested an anti-proliferative activity caused by Ad/IFN-*λ*1 or Ad/IFN-*λ*2 infection and found that YES-2 and T.Tn cells were sensitive to Ad/IFN-*λ*-mediated growth suppression, whereas TE-1 and YES-6 cells were insensitive (Figure 2C). Het-1A cells were also insensitive to the growth inhibition and Ad/LacZ did not influence cell proliferation. We also confirmed the suppressive activity with the WST assay (Figure 2D). The viability of YES-2 and T.Tn cells was reduced with Ad/INF-*λ* infection, whereas that of TE-1, YES-6 and Het-1A was unchanged. The responsiveness to IFN-*λ*s was thus diversified in respective cells, as found in the cases with increased MHC class I expression, but resistant to growth inhibition. Increased class I expression levels as well as growth inhibition levels were greater with Ad/IFN-*λ*2 than with Ad/IFN-*λ*1, irrespective of cell types. The differential activity between IFN-*λ*1 and IFN-*λ*2 was probably due to the expressed IFN amounts, as the same amount of rIFN-*λ*1 and rIFN-*λ*2 equally inhibited the growth of YES-2 and T.Tn cells (Figure 2E).

### Apoptosis induction by Ad/IFN-*λ* infection

**Figure 3:**
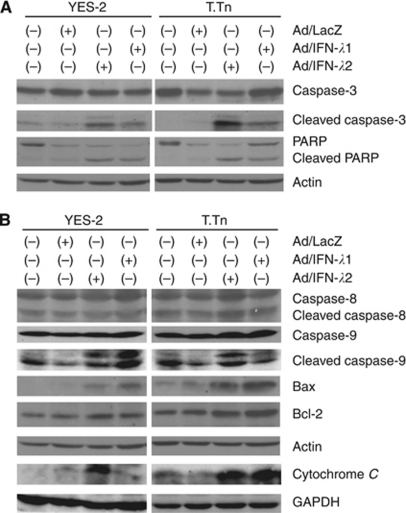
Expressions of apoptosis-linked proteins with Ad/IFN-*λ* treatments. YES-2 and T.Tn cells were infected with Ad (1000 MOI) and cultured for 72 h. Expressions of caspase-3, PARP, the respective cleaved forms (**A**), caspase-8, caspase-9, the respective cleaved forms, Bax, Bcl-2 and cytochrome *C* in a cytoplasmic fraction (**B**) were analysed with western blot analyses. Actin and GAPDH expression are shown as a loading control for total, cytoplasmic protein, respectively.

We investigated a possible mechanism of the growth suppression mediated by Ad/IFN-*λ*1 or Ad/IFN-*λ*2. Flow cytometrical analyses on cell cycle distributions showed an increased sub-G1 fraction in YES-2 and T.Tn cells as early as 48 h after the Ad infection and an elevated S-phase population in YES-2 cells (Table 1). We confirmed the S-phase increase in YES-2 cells with a nuclear PCNA staining, which was associated with DNA synthesis (Table 2). The positive cells increased with Ad/IFN-*λ*1 or Ad/IFN-*λ*2 infection in YES-2 but not in T.Tn cells. In contrast, insensitive TE-1 and YES-6 cells did not show any significant cell cycle changes. We then examined a possible apoptosis pathway with western blot analyses and showed that caspase-3 and PARP were cleaved after the infection with Ad/IFN-*λ*1 or Ad/IFN-*λ*2 in YES-2 and T.Tn cells (Figure 3A). Further analyses demonstrated that upregulation of cleaved caspase-8 and Bcl-2 expression was minimal, but expression levels of cleaved caspase-9, Bax and cytoplasmic cytochrome *C* increased in both cells after the infection (Figure 3B). The mitochondria fraction of cytochrome *C* remained unchanged (data not shown). These data collectively suggested that Ad/IFN-*λ*1 and Ad/IFN-*λ*2 primarily activated the mitochondria-mediated apoptosis pathway.

### Decreased tumourigenicity by Ad/IFN-*λ* infection

**Figure 4:**
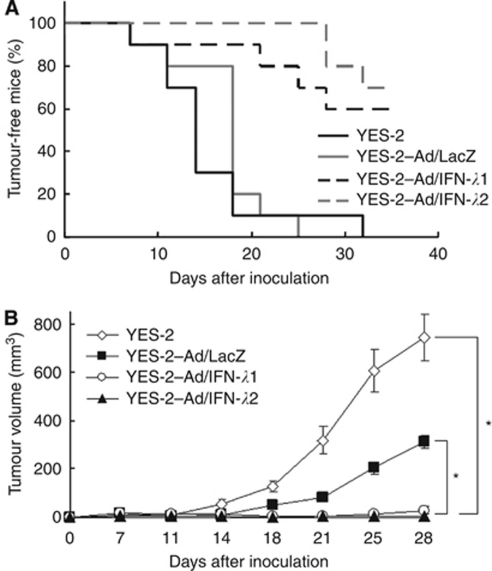
Decreased tumourigenicity of YES-2 cells by Ad/IFN-*λ*-mediated transduction. Uninfected YES-2 cells (YES-2), Ad/IFN–LacZ (1000 MOI)-infected YES-2 cells (YES-2–Ad/LacZ), Ad/IFN-*λ*1- or Ad/IFN-*λ*2 (1000 MOI)-infected YES-2 cells (YES-2-Ad/IFN-*λ*1, YES-2-Ad/IFN-*λ*2; 1 × 10^6^) were injected subcutaneously into nude mice (*n*=10). (**A**) Percentages of tumour-free mice. (**B**) The average tumour volumes with s.e. bars. ^*^P<0.01.

We examined whether transduction with Ad/IFN-*λ*1 or Ad/IFN-*λ*2 decreased tumourigenicity of the sensitive tumour cells. We subcutaneously inoculated YES-2 cells in nude mice after they were infected with Ad/IFN-*λ*1, Ad/IFN-*λ*2 or Ad/LacZ (Figure 4). The majority of the mice that were injected with Ad/IFN-*λ*1- or Ad/IFN-*λ*2-infected YES-2 cells did not develop the tumours, whereas all the mice that were injected with uninfected YES-2 or Ad/LacZ-infected YES-2 cells developed the tumours (Figure 4A). Percentages of tumour-free mice showed decreased tumourigenicity of Ad/IFN-*λ*-infected YES-2 cells (*P*<0.01), and the growth of developed YES-2 tumours infected with Ad/IFN-*λ* was also retarded compared with that of uninfected or Ad/LacZ-infected YES-2 tumours (Figure 4B). The growth of Ad/LacZ-infected YES-2 tumours was retarded compared with that of uninfected YES-2 tumours probably due to nonspecific cytotoxicity by Ad infection (Figure 4B).

### Anti-tumour effects produced by Ad/IFN-*λ*-infected fibroblasts

**Figure 5:**
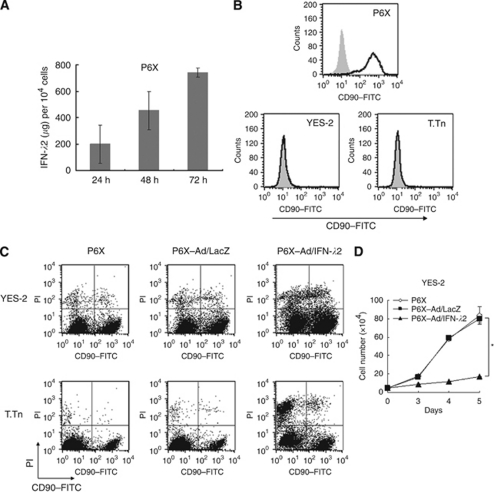
Tumour cell death induced by Ad/IFN-*λ*-infected P6X cells. (**A**) Interferon-*λ*2 in the culture supernatants of Ad/IFN-*λ*2 (1000 MOI)-infected P6X cells. (**B**) CD90 expression in P6X, YES-2 and T.Tn cells analysed with flow cytometry. Staining profiles with anti-CD90 Ab (black lines) or isotype-matched control Ab (gray shaded). (**C**) Representative staining profiles of CD90 and PI analysed with flow cytometry. Tumour cells and P6X cells, uninfected or infected with Ad/LacZ (3000 MOI; P6X–Ad/LacZ) or Ad/IFN-*λ*2 (3000 MOI; P6X–Ad/IFN-*λ*2), were cultured for 4 days. (**D**) Growth kinetics of YES-2 cells that were cultured with P6X cells, uninfected or infected with Ad/LacZ or Ad/IFN-*λ*2 (3000 MOI). The cell numbers were calculated based on CD90 and PI staining profiles as shown in Table 3. Means and s.e. bars are shown (*n*=3). ^*^P<0.05.

We explored a possible therapeutic strategy with IFN-*λ*-insensitive non-tumourous cells that can deliver IFN-*λ*s to target tumours. The P6X fibroblasts, lacking IFN-*λ* receptor complexes (Figure 2A), were insensitive to growth inhibition by Ad/IFN-*λ*2 (data not shown) but can secrete IFN-*λ*2 after Ad/IFN-*λ*2 infection (Figure 5A). The fibroblasts can thereby be a carrier to release IFN-*λ*2 into the vicinity. The IFN-*λ*2 production was greater in P6X cells than in TE-1 or YES-2 cells, which could be partly due to greater infectivity of P6X cells (Figure 1A). The other possible reasons, however, remained uncharacterised. We investigated whether IFN-*λ*2 released from Ad/IFN-*λ*2-infected P6X cells induced cell death of YES-2 or T.Tn cells that were co-cultured. We used CD90 molecules as a marker to distinguish between P6X cells (CD90^+^) and carcinoma cells (CD90^−^; Figure 5B). The oesophageal carcinoma cells were cultured with P6X cells that were uninfected or infected with Ad/LacZ or Ad/IFN-*λ*2, and were analysed for the cell death with flow cytometry (Figure 5C). The staining profiles with anti-CD90 Ab and PI showed that the CD90^−^/PI^+^ fraction and a ratio of the CD90^−^PI^+^ populations to all the CD90^−^ cells increased when the tumour cells were cultured with Ad/IFN-*λ*2-infected P6X cells compared with the co-culture cases with uninfected or Ad/LacZ-infected P6X cells (Table 3). We also estimated YES-2 cell numbers on the basis of the flow cytometrical data and found that the cell proliferation was retarded in the presence of Ad/IFN-*λ*2-infected but not Ad/LacZ-infected P6X cells (Figure 5D). These data collectively showed that both YES-2 and T.Tn cells were killed by IFN-*λ*2 secreted from P6X cells and subsequently the cell growth was inhibited. Interestingly, the percent ratio of CD90^+^/PI^+^ fraction to all the CD90^+^ cells also increased. It could be partly due to nonspecific Ad toxicities to P6X cells, but further investigations are required as P6X cells were insensitive to rIFN-*λ*2 (data not shown).

**Figure 6:**
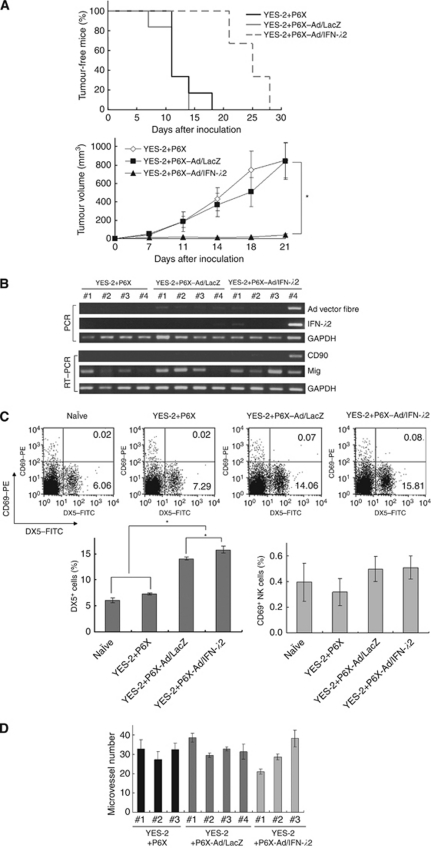
Anti-tumour effects produced by P6X cells infected with Ad/IFN-*λ*2. (**A**) YES-2 cells mixed with P6X cells, uninfected (P6X) or infected with Ad/IFN–LacZ (1000 MOI; P6X–Ad/LacZ) or Ad/IFN-*λ*2 (1000 MOI; P6X–Ad/IFN-*λ*2), were inoculated into nude mice (*n*=6). Percentages of tumour-free mice and average tumour volumes with s.e. bars are shown. (**B**) Amplification of the type 35 Ad fibre-knob region and IFN-*λ*2-encoding sequences with PCR, and the gene expression of *CD90* and *Mig* analysed by RT–PCR. *Glyceraldehyde-3-phosphate dehydrogenase* was used as respective controls. Samples were derived from day-21 tumours (*n*=4) as indicated. (**C**) DX5^+^ and CD69^+^ cells in spleen of naïve mice and the mice injected with mixed cells (day 10). Representative flow cytometrical data and percentages of DX5^+^CD69^−^ and DX5^+^CD69^+^ fractions are shown. Graphs show average percentages of DX5^+^ cells and CD69^+^ cells among DX5^+^ populations with s.e. bars (*n*=3). (**D**) CD31^+^ microvessel numbers per field ( × 10 magnification) based on immunohistochemical stainings of day-21 tumours. Three sections per each sample (#1-#4) were examined for the microvessel numbers, and the means with s.e. bars are shown. ^*^P<0.01.

We examined a possibility that P6X cells expressing IFN-*λ*2 inhibited the growth of YES-2 tumours (Figure 6A). We subcutaneously inoculated a mixed population of YES-2 cells and P6X cells that were uninfected or infected with Ad/LacZ or Ad/IFN-*λ*2. As P6X cells did not form tumours in nude mice (data not shown), any developed tumours were derived from YES-2 cells. The tumour development was delayed when YES-2 cells were mixed with Ad/IFN-*λ*2-infected P6X cells compared with the cases of uninfected or Ad/LacZ-infected P6X cells (*P*<0.01). The tumour growth was also retarded when YES-2 cells were mixed with Ad/IFN-*λ*2-infected P6X cells in comparison with the other cases. These data thus demonstrated that Ad/IFN-*λ*2-infected P6X cells suppressed the growth of co-injected YES-2 tumours.

### Mechanisms of anti-tumour effects *in vivo*

We analysed mechanisms involved in the anti-tumour effects produced by IFN-*λ*2-secreting P6X cells. We firstly examined persistency of P6X cells in YES-2 tumours developed in nude mice. PCR analyses with genomic DNA from day-21 tumours showed that the type 35 Ad fibre-knob region and the IFN-*λ*2 sequences were not amplified, except one tumour sample derived from YES-2 tumours mixed with Ad/IFN-*λ*2-infected P6X cells (Figure 6B). Reverse transcription PCR analysed with RNA from the day-21 tumours detected CD90 expression only in the same sample. These data suggest that most of P6X cells did not survive in nude mice as late as day 21. Serum concentrations of IFN-*λ*s were also undetectable on days 2, 6 and 10, suggesting that the effects of IFN-*λ*2 were not systemic but local.

We examined possible activation of NK cells with flow cytometry. Human IFN-*λ*s can activate murine IFN-*λ*s signalling pathways, as they are cross-reactive with each other (Lasfar *et al*, 2006). Spleen cells of the mice on day 10 were examined for the expression of DX5, a pan-NK cell marker, and CD69, an activated NK cell marker (Figure 6C). The numbers of DX5^+^ NK cells increased in spleens of the mice that received Ad-infected P6X cells compared with naïve mice and the mice injected with uninfected P6X cells. The NK cell numbers were also greater in the mice administered with Ad/IFN-*λ*2-infected P6X cells than in those with Ad/LacZ-infected cells. The percentages of CD69^+^ cells in DX5^+^ NK cells, however, were not different among spleens from naïve mice and the mice administered with P6X cells, uninfected or infected with Ad/LacZ or Ad/IFN-*λ*2. These data showed that inoculation of Ad infected cells increased NK cell populations and secreted IFN-*λ*2 enhanced the increase, but both Ad infection and IFN-*λ*2 did not activate NK cells. We also examined the CD69^+^ populations of B220^+^ B cells and CD11b^+^ macrophages, as CD69 is also an activation marker of them. The flow cytometrical analyses showed that ratios of CD69^+^ cells in B220^+^ or CD11b^+^ cells were not different among any four groups (data not shown).

We investigated a possible involvement of anti-angiogenesis in the anti-tumour effects. Immunohistochemical stainings of day-21 tumour specimens revealed that the numbers of CD31^+^ microvessels were not different among three groups, YES-2 tumours mixed with P6X cells, uninfected or infected with Ad/IFN-*λ*2 or Ad/LacZ (Figure 6D). Reverse transcription PCR analyses demonstrated that expression levels of the *Mig* gene, a potent inhibitor of angiogenesis, were not different among the groups (Figure 6B). These data collectively suggest that anti-angiogenesis did not contribute to the anti-tumour effects produced by Ad/IFN-*λ*2-infected P6X cells.

## Discussion

In this study, we investigated anti-tumour effects produced by Ad-expressing human *IFN-λ* genes and demonstrated that transduction of oesophageal carcinoma with the Ad induced the tumour cell death and inhibited the subsequent tumour growth. Fibroblasts, negative for the IFN-*λ* receptors, suppressed the growth of co-cultured or co-injected oesophageal carcinoma *in vitro* and *in vivo* when the fibroblasts were transduced with the *IFN-λ* gene. The IFN-*λ*s have multiple biological functions, which are comparable to those of type I IFNs as intracellular signal pathways of two IFN types seem to be related with each other (Kotenko *et al*, 2003; Li *et al*, 2009). The functions of IFN-*λ*s *in vivo*, however, have not been well investigated, although a few studies reported that growth of tumours expressing the murine *IFN-λ* genes was retarded in mice (Lasfar *et al*, 2006; Sato *et al*, 2006; Numasaki *et al*, 2007). Moreover, precise mechanisms of the growth-suppressive activity had not been characterised. Our previous study showed that growth of oesophageal carcinoma cells was suppressed by rIFN-*λ*1 due to either apoptosis induction or G1 phase arrest (Li *et al*, 2010). We thereby constructed fibre-modified Ad/IFN-*λ*1 and Ad/IFN-*λ*2 to investigate the growth inhibitory activity and demonstrated the cytotoxic effects on oesophageal carcinoma cells. To our knowledge, this is the first study to show that Ad expressing the *IFN-λ* gene produced anti-tumour effects and suggested that the Ad could be an agent for cancer treatments.

We previously analysed the susceptibility of a panel of oesophageal carcinoma cells to human rIFN-*λ*1 and showed that the IFN-*λ*1-mediated growth inhibition was dependent on the cells (Li *et al*, 2010). The present data showed that YES-2 and T.Tn cells, sensitive to rIFN-*λ*1, were susceptible to Ad/IFN-*λ*1- and Ad/IFN-*λ*2-induced growth inhibition, and rIFN-*λ*1-insensitive TE-1 and YES-6 cells were resistant to Ad/IFN-*λ*. In contrast to the growth susceptibility, transduction with Ad/IFN-*λ*1 or Ad/IFN-*λ*2 upregulated MHC class I antigen expressions in all the cells tested, demonstrating that signal pathways responsible for the growth inhibition have cell-type specificity. In the present study, we showed that biological activities induced by Ad/IFN-*λ*2, the class I antigen upregulation and the growth inhibition, were stronger than those by Ad/IFN-*λ*1. This is not due to property differences between IFN-*λ*1 and IFN-*λ*2 but probably due to differential productivity of IFN-*λ*s. The RT–PCR results showed that the oesophageal carcinoma cells expressed only the isoform 1 receptor (accession number: NP_734464) among the three IL-28R*α* isoforms (data not shown; Kotenko *et al*, 2003; Sheppard *et al*, 2003), which ruled out a possibility that potential difference of the ligand-binding affinity among the isoforms influences activation levels of the signal transduction. Northern blot analysis also showed that expression levels of the *IL-28Rα* gene, encoding a ligand-binding chain, were not correlated with the biological activity levels (data not shown). It is currently unknown why the efficacy of IFN production was better with Ad/IFN-*λ*2 than with Ad/IFN-*λ*1. The differential efficacy could be due to enhanced stability or decreased degradation of IFN-*λ*2 compared with IFN-*λ*1. Interestingly, Meager *et al* (2005) suggested that IFN-*λ*1 was stronger than other subtypes in anti-viral responses, but the mechanism remained uncharacterised.

Type I IFNs induced apoptosis in malignant cells through two major pathways, the death receptor-mediated and the mitochondria-mediated pathways (Chawla-Sarkar *et al*, 2003). In contrast, detailed pathways activated by type III IFNs have not been well investigated, although previous studies showed caspase-3, 8 and 9 activations upon IFN-*λ* treatments (Zitzmann *et al*, 2006; Li *et al*, 2008). The present study showed that Ad/IFN-*λ* activated the mitochondria-mediated rather than the death receptor-mediated apoptosis because we detected release of cytochrome C into the cytoplasm and Bax upregulation but minimal caspase-8 cleavage. It is difficult to compare the molecular expression levels between YES-2 and T.Tn cells, as YES-2 cells were susceptible to IFN-*λ* more than T.Tn cells and Ad/IFN-*λ*2 produced more potent activities than Ad/IFN-*λ*1. Nevertheless, the present western blot analyses showed that differential upregulated expressions of the apoptosis-linked molecules were in general concordant with the levels of Ad-induced growth suppression, except the Bax and the cleaved caspase-9 levels in YES-2 cells. These data collectively suggest that the cell-type specific susceptibility to IFN-*λ*s could be attributable to induction levels of the mitochondria-mediated apoptosis. It is crucial to differentiate IFN-*λ*s-sensitive cells from non-responders by using a possible biomarker for the potential clinical application. We also noticed that Ad/IFN-*λ* infection induced S-phase arrest as well as increased sub-G1 populations in YES-2 cells, but the mechanisms responsible for the S-phase arrest are currently unknown. Type I IFNs treatment can induce the S-phase arrest, followed by apoptosis (Murata *et al*, 2006; Vitale *et al*, 2006), but detailed information on the cell cycle arrest has not been reported.

We examined anti-tumour effects produced by Ad/IFN-*λ* in *in vivo* settings. Transduction of YES-2 cells with either Ad/IFN-*λ*1 or Ad/IFN-*λ*2 reduced the tumourigenicity, and the growth of developed tumours was also retarded. We further investigated a possible cell-mediated delivery of IFN-*λ*s with IL-28R*α*-negative immortalised fibroblasts, which did not develop tumours in nude mice. The fibroblasts infected with Ad/IFN-*λ*2 induced apoptosis of YES-2 and T.Tn cells that were co-cultured and suppressed growth of YES-2 tumours that were co-injected. The anti-tumour effects *in vivo* were also evidenced by delayed tumourigenicity of YES-2 cells. On the other hand, a direct intratumoural injection of Ad/IFN-*λ*1 or Ad/IFN-*λ*2, or injection of Ad/IFN-*λ*2 or Ad/IFN-*λ*2-infected P6X cells into established YES-2 tumours produced little anti-tumour effects (data not shown), which could be due to inefficient Ad-mediated transduction efficacy and Ad retention at the tumour sites or due to a poor migration activity of the transduced P6X cells into YES-2 tumours. These results rather showed limitations of Ad/IFN-*λ*-induced effects *in vivo* and fibroblasts-mediated delivery of IFN-*λ*s. Nevertheless, cell-mediated delivery of cytokines has been tested for its potential therapeutic effects on a certain types of cancer (Fritz and Jorgensen, 2008), and type I IFN delivery by stem/progenitor cells has achieved inhibition of tumour growth and metastasis (Studeny *et al*, 2004). The delivery system has several advantages in contrast to intratumoural injection of Ad vectors. The cell-mediated secretion can maintain a constant concentration of the released substance at a local milieu and probably induce immune responses against the exogenous substance to a less extent compared with the case of Ad vector administration, especially when the cells are of a syngeneic origin. Mesenchymal stem cells can be a suitable vehicle, as they have a propensity of migrating into tumours and constituting tumour stroma (Hamada *et al*, 2005). The Ad bearing the type-35-derived fibre-knob region infected mesenchymal stem cells as well as fibroblasts much better than type 5 Ad (Mizuguchi *et al*, 2005). The Ad/IFN-*λ*1 and Ad/IFN-*λ*2 with the fibre-knob modification are thereby appropriate vectors for cell-mediated gene therapy.

It could be crucial whether normal oesophageal epithelia are sensitive to IFN-*λ*1 and IFN-*λ*2 in the case of the clinical application. The present study showed that Het-1A, currently available non-transformed immortalised cells, upregulated the MHC class I expression by Ad/IFN-*λ* infection but were insensitive to the growth inhibitory actions. The present data suggest that IFN-*λ*-mediated therapies do not damage normal oesophageal tissues in the vicinity of the tumours. In contrast, IFN-*α* treatments induced growth inhibition of Het-1A cells as well as upregulation of the class I expressions (Li *et al*, 2010). Moreover, the expression of IFN-*λ* receptors complexes is relatively restricted in a tissue-specific manner (Sommereyns *et al*, 2008), which may circumvent possible systemic toxicities of IFN-*λ*s, in contrast to type I IFNs whose receptors are ubiquitously expressed (Pestka *et al*, 2004; Li *et al*, 2010). The tissue-type difference of the receptor expression can be an advantage of type III over type I IFNs. We, however, need to examine normal oesophageal epithelia to demonstrate that oesophagus is in fact resistant to the IFN-*λ*s-mediated cell killing. Moreover, precise analyses on the receptor distribution *in vivo* are required. A possible drawback of the cell-mediated IFN-*λ* delivery is the cytotoxicity to carrier cells as found in the co-culture experiments with P6X cells and YES-2 cells. The PI^+^ population of P6X cells was greater with Ad/IFN-*λ*2 infection than with Ad/LacZ infection. The Ad infection and/or secreted IFN-*λ*2 could induce the IL-28R*α* expression and subsequently P6X cells might become sensitive to IFN-*λ*2-induced cell death. We need further investigations about the induction of IFN-*λ* receptor complexes after viral infections and IFN treatments.

We examined mechanisms of anti-tumour effects by IFN-*λ*2 in the experimental *in vivo* model and showed that NK cell activity and anti-angiogenesis did not have a major role in the anti-tumour effects. As human IFN-*λ* can activate murine IFN-*λ* receptor complexes and vice versa (Lasfar *et al*, 2006), unchanged numbers of CD69^+^ NK cells and CD31^+^ cells were not due to the species difference. The most probable mechanism is that cell death of the oesophageal carcinoma was directly caused by IFN-*λ*2, although a murine immune system rejected most of the P6X cells. In contrast, previous studies reported that immune responses activated by IFN-*λ*2 produced anti-tumour effects (Sato *et al*, 2006; Numasaki *et al*, 2007). Transduction of murine colon and fibrosarcoma with the *IFN-λ2* gene activated NK cells and cytotoxic T cells, and consequently achieved anti-tumour effects against the tumours in experimental animal models. Lasfar *et al* (2006) however, failed to elicit protective immunity against IFN-*λ*2-producing melanoma, although the IFN-*λ*2-secreting melanoma cells were rejected. These studies collectively suggest that IFN-*λ* can achieve anti-tumour effects through multiple mechanisms depending on a tumour model used.

In conclusion, this study for the first time demonstrated a possible clinical application of Ad-mediated IFN-*λ* that induced the mitochondria-mediated apoptosis in oesophageal carcinoma cells. Precise mechanisms responsible for the anti-tumour actions remain to be elucidated, but this study revealed a therapeutic potential of cell-mediated delivery of IFN-*λ* for cancer therapy.

## Figures and Tables

**Table 1:** Cell cycle distribution after Ad/IFN-*λ* treatments

		**Cell cycle distribution (%)** b			
**Cell**	**Treatment** a	**Sub-G1**	**G0/G1**	**S**	**G2/M**
TE-1	(−)^c^	0.4±0.1	58.5±0.5	12.9±0.8	28.8±1.4
	Ad/LacZ	0.6±0.1	45.8±0.8	18.3±0.4	35.7±1.2
	Ad/IFN-*λ*1	0.5±0.1	49.3±0.4	17.7±0.6	32.8±0.9
	Ad/IFN-*λ*2	0.4±0.1	52.6±0.2	16.4±0.6	30.9±0.4
					
YES-2	(−)	4.3±0.3	42.3±0.9	18.8±0.4	34.5±0.8
	Ad/LacZ	7.1±0.7	35.2±3.3	16.4±0.5	41.1±2.7
	Ad/IFN-*λ*1	11.9±0.8^d^	34.0±1.1	22.2±0.2^d^	31.6±2.5
	Ad/IFN-*λ*2	18.8±1.5^d^	30.4±1.2	21.3±0.5^d^	29.2±0.4
					
YES-6	(−)	1.0±0.2	58.9±0.9	12.0±0.3	28.4±0.5
	Ad/LacZ	1.0±0.1	55.8±0.7	13.6±0.3	29.9±1.0
	Ad/IFN-*λ*1	1.2±0.1	53.1±0.7	15.0±0.5	31.0±0.8
	Ad/IFN-*λ*2	1.0±0.1	57.4±1.0	15.6±0.7	26.4±1.2
					
T.Tn	(−)	2.9±0.1	59.9±0.6	14.6±0.2	23.0±0.4
	Ad/LacZ	6.1±0.3	55.0±0.6	16.1±0.3	23.4±0.7
	Ad/IFN-*λ*1	15.0±0.2^d^	49.4±0.3	16.8±0.4	19.6±0.3
	Ad/IFN-*λ*2	27.2±0.5^d^	37.2±0.1	16.9±0.4	19.9±0.6

Abbreviations; Ad=adenoviruses; IFN=interferon; LacZ=*β*-galactosidase; MOI=multiplicity of infection.

a Cells were infected with Ad (3000 MOI) and cultured for 72 h.

b Cell cycle profiles were analysed with flow cytometry and the data are shown in mean percentages with s.e.s (*n*=3).

c (−), not infected.

d *P*<0.01, comparing between Ad/IFN-*λ*-infected and untreated or Ad/LacZ-infected cells.

**Table 2:** Nuclear PCNA-positive cells after Ad/IFN-*λ* treatments^a^

**Cells**	**Treatment**	**PCNA-positive cells (%**±**s.e.)**
T.Tn	(−)^b^	0.23±0.15
	Ad/LacZ	1.4±0.55
	Ad/IFN-*λ*1	0.86±0.26
	Ad/IFN-*λ*2	0.84±0.12
		
YES-2	(−)	1.4±0.59
	Ad/LacZ	1.2±0.41
	Ad/IFN-*λ*1	3.3±0.49^c^
	Ad/IFN-*λ*2	4.0±0.89^c^

Abbreviations; Ad=adenoviruses; IFN=interferon; LacZ=*β*-galactosidase; MOI=multiplicity of infection; PCNA=proliferation cell nuclear antigen.

a Cells were infected with Ad (1000 MOI), cultured for 72 and nuclear PCNA-positive cells were microscopically counted among 150 cells in total. The data are shown in mean percentages with s.e.s (*n*=3).

b (−), not infected.

c *P*<0.05, comparing between Ad/IFN-*λ*-infected and untreated or Ad/LacZ-infected cells.

**Table 3:** Tumour cell death induced by P6X cells infected with Ad/IFN-*λ*2^a^

	**YES-2 cells cultured with**			**T.Tn cells cultured with**		
**Sub-population**	**P6X**	**Ad/LacZ**–**P6X**	**Ad/IFN-*λ*2**–**P6X**	**P6X**	**Ad/LacZ**–**P6X**	**Ad/IFN-*λ*2**–**P6X**
CD90^−^PI^−^	64.6±0.5	66.1±0.1	40.0±0.6	84.4±0.5	86.1±0.7	53.2±0.4
CD90^−^PI^+^	2.1±0.1	2.4±0.1	6.5±0.1^d^	1.1±0.1	1.3±0.1	20.8±0.1^d^
CD90^+^PI^−^	32.6±0.5	30.1±0.1	48.7±0.4	14.4±0.5	12.2±0.8	23.2±0.2
CD90^+^PI^+^	0.8±0.1	1.4±0.1	4.8±0.2^d^	0.2±0.1	0.4±0.1	2.8±0.2^d^
CD90^−^PI^+^ /CD90^−b^	3.0±0.1	3.5±0.1	13.9±0.4^d^	1.2±0.1	1.5±0.2	28.1±0.5^d^
CD90^+^PI^+^ /CD90^+c^	2.3±0.1	4.4±0.1	8.9±0.2^d^	1.3±0.1	3.5±0.5	10.8±0.6^d^

Abbreviations; Ad=adenoviruses; IFN=interferon; LacZ=*β*-galactosidase; MOI=multiplicity of infection; PI=propidium iodide.

a YES-2 or T.Tn cells were cultured with P6X cells, uninfected or infected with Ad/LacZ or Ad/IFN-*λ*2 (3000 MOI), at a ratio of 1 : 1 (YES-2) or 2 : 1 (T.Tn). CD90 and PI staining profiles were analysed with flow cytometry after 96 h. Data are shown in mean percentages with s.e.s (*n*=3).

b Percent ratio of CD90^−^PI^+^ populations to all the CD90^−^ cells.

c Percent ratio of CD90^+^PI^+^ populations to all the CD90^+^ cells.

d *P*<0.01, comparing between Ad/IFN-*λ*2-infected P6X and uninfected or Ad/LacZ-infected P6X.
